# Removal of reproductive suppression reveals latent sex differences in brain steroid hormone receptors in naked mole-rats, *Heterocephalus glaber*

**DOI:** 10.1186/s13293-015-0050-x

**Published:** 2015-12-10

**Authors:** Ashlyn Swift-Gallant, Kaiguo Mo, Deane E. Peragine, D. Ashley Monks, Melissa M. Holmes

**Affiliations:** Department of Psychology, University of Toronto Mississauga, 3359 Mississauga Road, Mississauga, ON L5L 1C6 Canada; Department of Cell and Systems Biology, University of Toronto, 100 St. George Street, Toronto, ON M5S 3G3 Canada; Department of Ecology and Evolutionary Biology, University of Toronto, 25 Willcocks Street, Toronto, ON M5S 3B2 Canada

**Keywords:** Androgen receptor, Aromatase, Estradiol, Estrogen receptor, Eusocial, Naked mole-rat, Progesterone receptor, Reproductive suppression, Sex difference, Testosterone

## Abstract

**Background:**

Naked mole-rats are eusocial mammals, living in large colonies with a single breeding female and 1–3 breeding males. Breeders are socially dominant, and only the breeders exhibit traditional sex differences in circulating gonadal steroid hormones and reproductive behaviors. Non-reproductive subordinates also fail to show sex differences in overall body size, external genital morphology, and non-reproductive behaviors. However, subordinates can transition to breeding status if removed from their colony and housed with an opposite-sex conspecific, suggesting the presence of latent sex differences. Here, we assessed the expression of steroid hormone receptor and aromatase messenger RNA (mRNA) in the brains of males and females as they transitioned in social and reproductive status.

**Methods:**

We compared in-colony subordinates to opposite-sex subordinate pairs that were removed from their colony for either 1 day, 1 week, 1 month, or until they became breeders (i.e., produced a litter). Diencephalic tissue was collected and mRNA of androgen receptor (*Ar*), estrogen receptor alpha (*Esr1*), progesterone receptor (*Pgr*), and aromatase (*Cyp19a1*) was measured using qPCR. Testosterone, 17β-estradiol, and progesterone from serum were also measured.

**Results:**

As early as 1 week post-removal, males exhibited increased diencephalic *Ar* mRNA and circulating testosterone, whereas females had increased *Cyp19a1* mRNA in the diencephalon. At 1 month post-removal, females exhibited increased 17β-estradiol and progesterone. The largest changes in steroid hormone receptors were observed in breeders. Breeding females had a threefold increase in *Cyp19a1* and fivefold increases in *Esr1* and *Pgr*, whereas breeding males had reduced *Pgr* and increased *Ar*.

**Conclusions:**

These data demonstrate that sex differences in circulating gonadal steroids and hypothalamic gene expression emerge weeks to months after subordinate animals are removed from reproductive suppression in their home colony.

## Background

Sexual differentiation of the vertebrate brain and behavior is mediated in large part by gonadal steroid hormones acting in early development. In mammals, sexually differentiated hormone production begins at the earliest stages of gonadal development and produces marked sexual dimorphism in brain and genitals by early post-natal life (for review, see [1, 2]).

Naked mole-rats (*Heterocephalus glaber)* do not appear to fit this framework of sexual differentiation: most individuals fail to show traditional sex differences in neuroanatomy or behavior, although these features vary based on reproductive and social status [3–6]. Naked mole-rats are eusocial rodents, living in large colonies with a strict reproductive and social hierarchy: a single female and 1–3 males reproduce and are socially dominant, while others in the colony are subordinate and usually remain reproductively suppressed for the duration of their life [7–9]. The social status within a colony is remarkably stable; however, subordinates can achieve breeder status after loss of a breeder due to death or removal from the colony. In their natural environment, subordinate naked mole-rats can—though rarely—leave their natal colony to find an opposite sex partner and establish their own colony [10, 11]. In captivity, subordinates only undergo sexual maturation if they are separated from their colony [5, 12, 13] or if the established breeders die or are removed [14, 15].

Regardless of the specific circumstances in which subordinates become breeders, they only perform sexual behaviors and show mammalian typical sex differences in gonadal steroid hormones as they transition to breeding status. Subordinate females have lower urinary and plasma progesterone (P) levels as well as plasma luteinizing hormone (LH) concentrations compared to breeder females [16–18]. Within 3 days of separation from their natal colony, subordinate females show increases in urinary P levels, and within 7 days, they develop a perforated vagina [16]. Similarly, male subordinates have lower urinary testosterone (T) compared to male breeders in the colony [14, 15, 19], and urinary T levels and plasma LH increase when subordinate males are removed from their colony [20].

Subordinates and breeders also differ in the neural structures associated with sexual behaviors. However, where sex differences in the nervous system are found in most mammals, status differences are more prominent in the naked mole-rat. For example, breeders, regardless of sex, have larger regional volume in various reproductively relevant hypothalamic structures [4], as well as increased motoneuron soma size in Onuf’s nucleus [3]. Furthermore, androgen receptor (AR) and oxytocin immunoreactivity are influenced by social status more than sex [6, 21, 22].

Collectively, these data reveal a complex interplay between sex and social status in neuroendocrine function in these highly social mammals. To investigate how an apparent reduction or lack of neural sex differences can be associated with typical sex differences in gonadal function seen in breeders, the present study evaluated the time course of changes in gene expression of gonadal steroid hormone receptors and aromatase in brain, as well as associated circulating hormones. Thus, we quantified diencephalic levels of messenger RNA (mRNA) expression of androgen receptor (*Ar*), estrogen receptor alpha (*Esr1*), progesterone receptor (*Pgr*), and aromatase (*Cyp19a1*) and measured circulating T, 17β-estradiol (E2), and P, as animals transitioned from subordinate to breeding status. We hypothesized that sex differences in steroid hormone receptors would be associated with the differences in circulating gonadal steroids that emerge in breeders, specifically predicting that females would show increases in *Esr1* and *Pgr* while males would show increases in *Ar*.

## Methods

### Animals and housing

Sixty-two animals from 11 colonies were used in this experiment. Opposite-sex pairs of animals were established either 1 day (24 h), 1 week (7 days), or 1 month (30 days) prior to tissue collection (6 pairs per time point). Breeders were animals that were paired until a litter was produced (*n* = 5 pairs) and were collected 30 days after birth of their first litter. In naked mole-rats, gestation is between 65 and 74 days long, and lactation ceases at approximately 28 days post-partum [5, 23]. Taken with the fact that none of the breeding females were pregnant at the time of tissue collection, it seems unlikely that their values reported below were affected by either pregnancy or lactation. Some animals were mis-sexed, resulting in same-sex pairs. Same-sex pairs were excluded from analyses; therefore, the final numbers were five opposite-sex pairs in the 1-day group, 1-week group, and breeder group and six opposite-sex pairs in the 1-month group. In addition, seven male and five female subordinate animals were selected from the colonies immediately prior to tissue collection.

The colonies were housed in polycarbonate cages connected by tubing, as previously described [24]. The paired animals were housed in single polycarbonate cages (*L* 43 × *W* 22 × *H* 21 cm). All animals were kept on a 12:12-light/dark cycle at 28–30 °C and had ad libitum access to a diet of sweet potato and wet protein mash (Harlan Laboratories, Inc). All procedures were approved by the University Animal Care Committee and adhered to institutional and federal guidelines.

### Tissue collection

The animals were weighed prior to being overdosed with Avertin (40 mg/100 g). The bloods were collected from the trunk, and the brains were extracted. The brains were bisected at midline in the sagittal plane, frozen in liquid nitrogen, and stored at −80 °C until RNA extraction. The blood samples were kept on wet ice until centrifuged, after which serum was collected and stored at −20 °C.

### mRNA expression

The complete diencephalon from the right hemisphere of each subject was dissected and transferred to a sterile tube; every attempt was made to collect the same amount of tissue for all animals. RNA extraction from tissues and complementary DNA (cDNA) was prepared as previously described [25]. For primer sequences, refer to Table 1. Each experimental group (1-day, 1-week, 1-month, and breeder) was run separately with the same-sex subordinates included as controls in each reaction. For each reaction, the expression of the test gene was first normalized to the expression of GAPDH and fold changes were calculated relative to a control group (i.e., same-sex subordinates) as previously described [25].

**Table 1:** Primer sequences

Gene	Primers
Androgen receptor (*Ar*)	Forward: CTGGAAGTGGCCTCAGAAAG Reverse: GCGCTGTCAGATATGGTTGA
Estrogen receptor alpha (*Esr1*)	Forward: GAGGACTTGGTCCTGGATGA Reverse: CCAGGGCCTTCAGTAAGACA
Progesterone receptor (*Pgr*)	Forward: CAGTTGGTCCCTCCACTGAT Reverse: TGCCTCTCGCCTAGTTGATT
Aromatase (*Cyp19a1*)	Forward: TCGTCCTGGTGACACTTCTG Reverse: GGCAGGTCATCCATCTCATT
Glyceraldehyde 3′ phosphate dehydrogenase (GAPDH)	Forward: CCAAGGTCATCCACGACAAT Reverse: ACGCTGGGATGATGTTCTG

### Hormone assays

 Total T was measured from serum using an enzyme-linked immunosorbent assay (ELISA) from Enzo Life Sciences (Cat. No. ADI-901-065), following the manufacturer’s guidelines. The serum was diluted 5× with an assay buffer. The assay sensitivity is 5.67 pg/mL, and the intra-assay coefficient of variation is less than 10 %. Cross-reactivity is 14.6 % for 19-hydroxytestosterone and 7.2 % for androstenedione but less than 1 % for any other hormone or metabolite.

P was measured using an ELISA kit from Cayman Chemical (Cat. No. 582601) according to the manufacturer’s protocol. The serum was diluted 10× with an assay buffer provided. The assay sensitivity is 10 pg/mL, and the intra-assay coefficient of variance is less than 12 %. Cross-reactivity is reported with 17β-estradiol (7.2 %), 5β-pregnan-3α-ol-20-one (6.7 %), pregnenolone (2.5 %), and less than 0.5 % with any other hormone or metabolite.

E2 was measured with an ELISA, following the manufacturer’s protocol (GenWay Biotech, Cat. No. GWB-37E590). The serum was diluted 10× with an assay buffer provided. The assay sensitivity is 3.94 pg/mL, and intra-assay coefficient of variation is less than 10 %. Cross-reactivity is reported with estrone-3-sulfate (4.9 %).

Absorbance for each ELISA plate was measured using a Synergy-HT-Bio-Tek plate reader and was read at 405 nm for T and P and 450 nm for E2, as per the manufacturer’s protocol. All samples were run in duplicate.

### Statistical analyses

For mRNA comparisons, we first compared the subordinate values across reactions and evaluated sex differences using a repeated measures ANOVA. We did not find any significant differences between the reactions nor between the sexes (see below), so we then calculated a mean value for each subordinate, for each gene, based on the different reactions. Next, in order to compare the fold change from the subordinate controls, we divided the mRNA value for each experimental subject by the same-sex subordinate group average for each gene. We then conducted a two-way group by sex ANOVA for each target gene. Two-way group by sex ANOVAs were also conducted on the measures of body weight, age, and hormone concentrations. For hormone concentrations, the averages of duplicate readings were corrected by the dilution factor prior to analysis. Tukey’s HSD was used for all post hoc analyses. SPSS version 21 was used for all statistical analyses, and significance was determined by the critical alpha level of <0.05.

## Results

### mRNA expression

When comparing subordinate values across the different reactions, there were no significant effects of sex or reaction on any gene examined. (*Ar*: reaction by sex interaction, *F* (3, 24) = 0.101, *p* = .959; main effect [ME] of reaction, *F* (3, 24) = 0.130, *p* = .942; and ME of sex, *F* (1, 8) = 0.007, *p* = .937. *Cyp19a1*: reaction by sex interaction, *F* (3, 24) = 0.089, *p* = .966; ME of reaction, *F* (3, 24) = 0.063, *p* = .979; and ME of sex, *F* (1, 8) = 0.117, *p* = .741. *Esr1*: reaction by sex interaction, *F* (3, 24) = 0.094, *p* = .962; ME of reaction, *F* (3, 24) = 0.042, *p* = .988; and ME of sex, *F* (1, 8) = 0.046, *p* = .836. *Pgr*: reaction by sex interaction, *F* (3, 24) = 0.053, *p* = .983; ME of reaction, *F* (3, 24) = 0.119, *p* = .948; and ME of sex, *F* (1, 8) = 0.055, *p* = .820). For further analyses, we therefore averaged subordinate mRNA values from all reactions and compared them to the other four groups (1-day, 1-week, 1-month, and breeder).

**Fig. 1:**
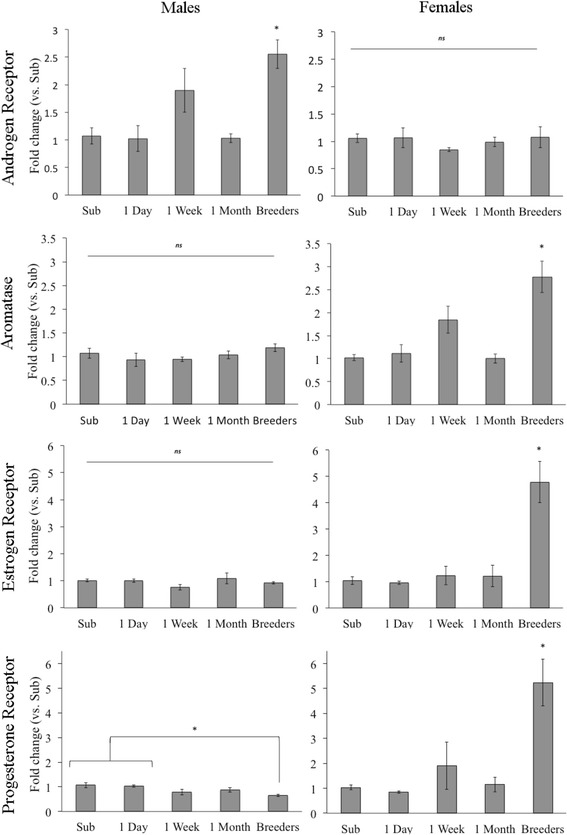
Mean fold change of diencephalic mRNA expression compared to subordinate animals ± standard error of the mean. *Ar* mRNA was significantly higher in male breeders compared to subordinate, 1-day, and 1-month, but not from 1-week, paired males. Females did not show changes in *Ar* mRNA across conditions, although *Cyp19a1* (aromatase) and *Esr1* mRNA were both increased in female breeders compared to 1-month, 1-week, 1-day, and subordinate females. Males did not show changes in *Esr1* or *Cyp19a1* mRNA. *Pgr* mRNA was decreased in male breeders compared to subordinates and 1-day paired males but did not differ from 1-week and 1-month paired males. Female breeders had significantly increased *Pgr* mRNA compared to all other female groups. *p < .05. *ns* non-significant

A significant interaction of group and sex was found for *Ar*, *F* (4, 43) = 6.398, *p* < .001, such that male breeders had significantly higher *Ar* mRNA levels than the subordinates, *p* = .002, 1-day males, *p* = .001, and 1-month males, *p* = .001, although they did not significantly differ from the 1-week males, *p* = .354. Conversely, the female groups did not differ in *Ar* mRNA expression (see Fig. 1). A significant main effect of sex, *F* (1, 43) = 16.76, *p* < .001, indicates that overall males have more *Ar* mRNA expression than females. Together, the results indicate changes in *Ar* mRNA in males, such that AR spikes at 1 week, followed by a decline at 1 month, and then again increases once breeder status is achieved.

A significant interaction of group and sex was also found for *Cyp19a1*, *F* (4, 43) = 8.712, *p* < .001; however, in this case, the male groups did not significantly differ from each other (all *p* > .05). The female breeders had significantly more *Cyp19a1* mRNA compared to the 1-month females, *p* < .001, 1-day females, *p* < .001, and subordinates, *p* < .001, and was marginally increased compared to the 1-week females, *p* = .058. A main effect of sex indicates that females had more *Cyp19a1* mRNA compared to males, *F* (1, 43) = 23.428, *p* < .001, and the main effect of group indicates that overall breeders had significantly more *Cyp19a1* mRNA compared to all other groups (see Fig. 1).

Similar to *Cyp19a1*, a group by sex interaction, *F* (4, 43) = 14.131, *p* < .001, indicates that female breeders have significantly more *Esr1* mRNA than all other female groups (1-month, 1-day, 1- week, and subordinate, all *p* < .001), in addition to a main effect of sex in which females have more *Esr1* mRNA than males, *F* (2, 43) = 20.51, *p* < .001. The male groups did not significantly differ from each other (see Fig. 1). In our sample, *Esr1* mRNA did not show any changes in females until after breeder status was achieved.

*Pgr* comparisons also revealed a significant group by sex interaction, *F* (4, 43) = 11.103, *p* < .001. The male breeders had significantly less *Pgr* mRNA compared to the subordinates, *p* = .015, and 1-day males, *p* = .026, but did not differ from the 1-month, *p* = .343, nor from the 1-week males, *p* = .829. Conversely, the female breeders had significantly more *Pgr* mRNA compared to all female groups (1-month, 1-day, 1-week, and subordinate, *p* < .01). The 1-month females did not differ from any group other than the female breeders, suggesting that *Pgr* does not increase in females until a litter is produced (see Fig. 1).

### Hormone assays

**Fig. 2:**
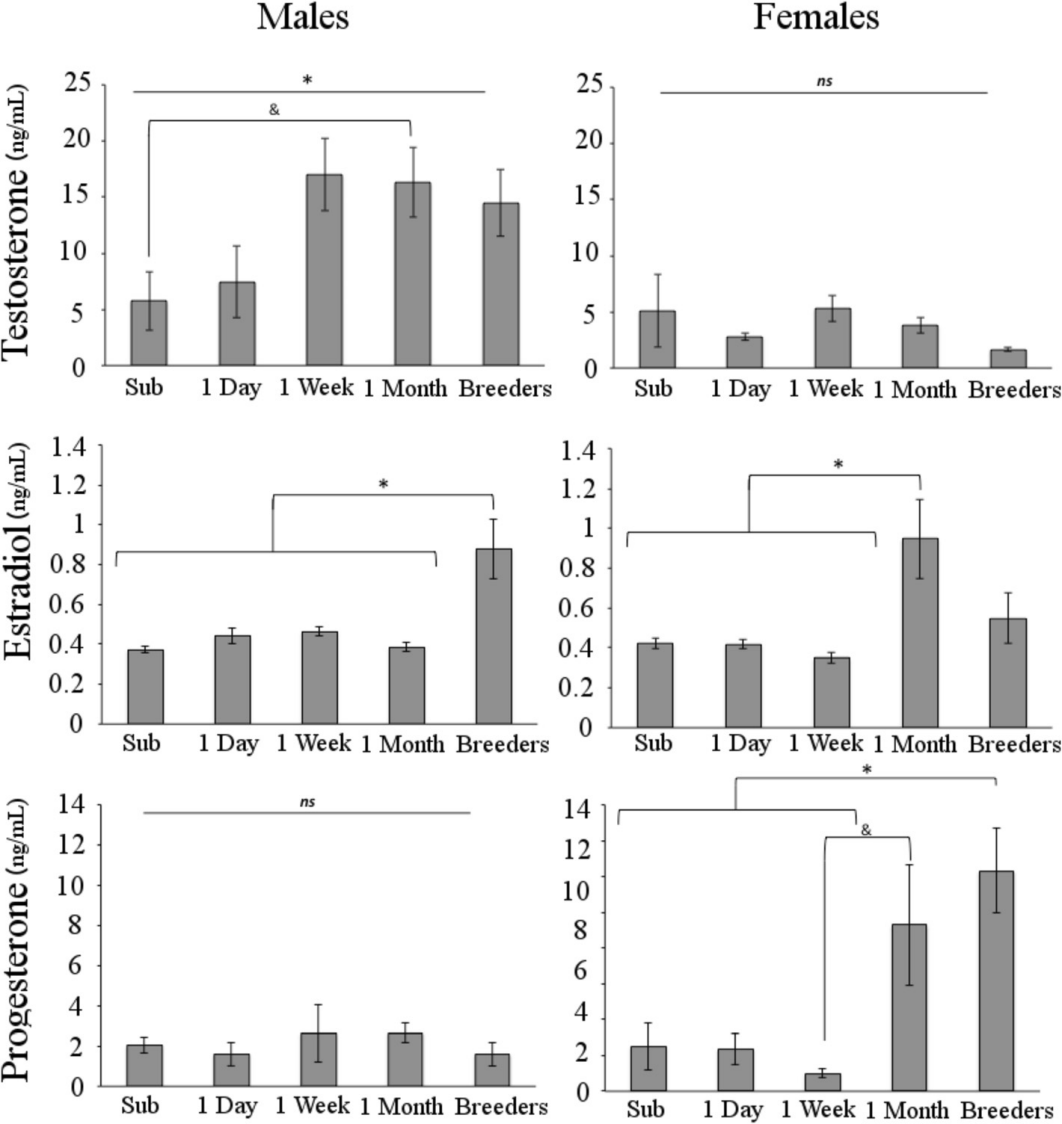
Mean hormone concentration (ng/mL) ± standard error of the mean. An interaction between sex and group indicates that males, but not females, had increased circulating testosterone following 1 week of pairing. Females had increased estradiol and progesterone at 1 month, although estradiol declined after becoming a breeder. Estradiol, but not progesterone, was increased in males that produced a litter. *p < .05; &*p* < .10. *ns* non-significant

T concentrations varied according to sex and group, *F* (4, 45) = 2.664, *p* = .044. The 1-month males had marginally more T compared to the subordinates, *p* = .091, whereas females did not show any differences across groups. Although other male groups are not significantly different from each other, there appears to be a trend for males to have an increase in T as early as 1 week after separation from the colony (see Fig. 2). A main effect of sex indicates that males have more T than females, *F* (1, 45) = 28.526, *p* < .001, and a main effect of group was trending, *F* (4, 45) = 2.215, *p* = .082, indicating a tendency for an increase in T over time away from the colony.

A sex by group interaction for E2 concentration, *F* (4, 43) = 6.896, *p* < .001, indicated that male breeders, but not female breeders, have increased E2 compared to the same-sex subordinate, *p* < .001; 1-day, *p* = .002, 1-week, *p* = .00, and 1-month groups, *p* < .001. Interestingly, the females showed an increase in E2 at 1 month, compared to the 1-week, *p* = .009, 1-day, *p* = .02, and subordinate groups, *p* = .024, but E2 declined once they produced a litter (see Fig. 2).

P concentrations also varied according to sex and group, *F* (4, 45) = 5.26, *p* = .001, such that female breeders, but not males, had increased P compared to the 1-week females, *p* = .01, 1-day, *p* = .027, and subordinate females, *p* = .03, but not the 1-month females, *p* = .835. There was a trend for the 1-month females to have more P than the 1-week females, suggesting that this hormone increases in females prior to producing a litter. A significant main effect of sex indicated that overall females have more P than males, *F* (1, 45) = 11.287, *p* = .002. A main effect of group, *F* (4, 45) = 4.909, *p* = .002, suggests that overall breeders have increased P compared to the 1-week, *p* = .02, 1-day, *p* = .024, and subordinate groups, *p* = .024, but not from the 1-month group, *p* = .967 (see Fig. 2).

### Body weight and age

The animals ranged in age from 12 to 38 months of age (*M* = 23.10, SD = 7.17). No significant differences in age were found between the groups, *F* (4, 42) = 1.46, *p* = .231, nor between the sexes, *F* (1, 42) = 0.631, *p =* .431. Further, no significant interaction was found between group and sex, *F* (4, 42) = 0.161, *p* = .957.

**Fig. 3:**
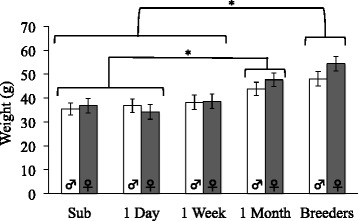
Group means ± standard error of the mean (SEM) for body weight at time of dissections. The breeders weighed significantly more than the animals in the subordinate, 1-day, and 1-week groups. The animals in the 1-month group weighed significantly more than the subordinates and 1-day group, but did not differ from the 1-week group or breeders. Subordinate, 1-day, and 1-week groups did not differ from each other. *p < .05

Body weight significantly differed between the groups, *F* (4, 41) = 5.31, *p* < .001, but not between sexes, *F* (1, 41) = 1.41, *p* = .316 (see Fig. 3), indicating that the breeders weighed more than the subordinates, *p* < .001; 1-day, *p* < .001, and 1-week group, *p* = .001. The 1-month group also significantly differed in weight from the subordinates, *p* = .009, and 1-week group, *p* = .008, but did not significantly differ from the 1-day group, *p* = .104, nor from the breeders, *p* = .348.

## Discussion

The present study demonstrates that sex-specific changes in neuroendocrine function are associated with the transition to reproductive status in naked mole-rats. We observed the emergence of latent sex differences in diencephalic gene expression and confirmed the emergence of sex differences in circulating gonadal steroids within a week after the animals are removed from reproductive suppression in their home colony. As discussed below, many of these neuroendocrine changes emulate typical sex differences observed in other species during puberty and reproduction, and these results may indicate the endocrine underpinnings of eusociality in naked mole-rats.

The first change we observed in the males was an increase in *Ar* mRNA expression that mirrored increased circulating T at 1 week post-removal from the colony. The increase in circulating T at this time point is consistent with previous reports in male naked mole-rats [12, 20] and suggests that T action via hypothalamic *Ar* is associated with the onset of sexual maturation in male naked mole-rats, analogous to other mammalian species. For example, in hamsters, rats, and ferrets, circulating T at puberty is required for male-typical sexual behavior, as well as to suppress female-typical sexual behavior (for review, see [26]). Interestingly, *Ar* mRNA was not consistently high after removal from the colony. At 1 month, *Ar* mRNA levels returned to baseline subordinate levels. Transient increases in *Ar* expression may reflect the termination of an initial phase of reproductive maturation in the breeder males. Alternatively, or additionally, reduced AR protein may be associated with a transition into a parental state. In birds, fish, humans, and other mammals, fathers-to-be exhibit decreases in T prior to the birth of their offspring [27–32], and we have previously reported reduced AR immunoreactivity in the breeding males compared to the subordinates [21, 22]. Decreases in T have been associated with the nurturing aspects of parenting rather than the defense of young/competitive aspects of parenting (for review, see [33]). However, none of our 1-month pairs were obviously pregnant at the time of tissue collection, and T levels remained high in the 1-month paired males. Future experiments evaluating behavior during peaks in *Ar* mRNA, as well as manipulating circulating T levels, will test both the functional significance of changes in gene expression and the consistency across species of the association between androgens and sexual maturation/mating behavior.

It warrants mention that the status difference in *Ar* mRNA expression we report here, with the breeding males having a 2.5-fold increase over the subordinate males, is in the opposite direction of status differences in AR protein that we have previously reported [21, 22]. Indeed, increased AR immunoreactivity would be predicted in breeders as androgens typically both stabilize AR protein and facilitate immunohistochemical detection by causing nuclear translocation. Three possibilities might explain this apparent discrepancy. First, our work with AR protein quantified immunoreactivity in particular brain regions whereas the current report does not have the same anatomical resolution. Second, our previous reports compared groups of subordinates and breeders without controlling for duration of pairing, number of litters/size of colony, presence of new litter, etc., whereas the breeders herein were all collected exactly 30 days after the birth of their first litter. Finally, patterns of AR protein and mRNA expression are not always complementary (for review, see [34]). This raises the possibility that, in this case, non-coordinate regulation of AR may be achieved via differences in mRNA translation. Although this is an uncommon mechanism, naked mole-rats have a ribosomal organization which is unique in eukaryotic organisms [35] and could very well have similarly unique regulation of protein translation. It will be important to directly quantify protein and mRNA in the same animals to test these alternatives.

In addition to the increase in *Ar* mRNA after the production of a litter, male naked mole-rats exhibit other neuroendocrine changes similar to new fathers from other species. For example, circulating E2 increased and *Pgr* mRNA decreased in new naked mole-rat fathers. In humans, an increase in E2 has been reported in men becoming fathers that lasted from the last month of pregnancy to the first month after pregnancy [28]. Reduced *Pgr* has also been associated with increased paternal behavior in mice: *Pgr*-knockout mice and pharmacological antagonism of *Pgr* increased parental care and decreased infanticide behavior without diminishing male-male aggression, whereas increased P has the opposite effect ([36]; for review, see [37]). Alternatively, it is possible that this small decrease in *Pgr* is not biologically functional. Future experiments could evaluate whether PR has a role similar to other species in male naked mole-rats.

In female naked mole-rats, the first observed endocrine change was an increase in circulating E2 and P 1 month post-removal from their colony. This increase may be indicative of sexual maturation and sexual receptivity. Estrogens are known to trigger, and P to reinforce, the events leading to ovulation in other rodent species (for review, see [38, 39]). E2 is also related to lordosis behavior in rodents, and P acting via progesterone receptor amplifies this behavior (for review, see [40]). Increases in *Esr1* and *Pgr* mRNA were not apparent in female naked mole-rats until after the production of a litter; thus, it is possible that increases in circulating E2 and P are responsible for the increase in diencephalic mRNA expression of the corresponding receptors. However, it is also possible that this increase in gene expression is related to new motherhood, as in other species, increases in *Esr1* are also associated with maternal care [41].

The timing of our increase in E2 and P at 1 month post-removal is later then we predicted based on reports of increased P in the urine of female naked mole-rats approximately 1 week after removal from the colony [12, 16]. This might reflect different hormone concentrations in urine versus blood, differences in assay sensitivity, or other methodological differences concerning sample collection. We collected the blood at a single time point whereas previous reports collected urine at multiple times per animal [12, 16]. Alternatively, the variability between experiments might reflect individual differences in the timing of reproductive maturation in subordinate naked mole-rats. New pairs of opposite-sex subordinates show substantial variability in the amount of time to produce their first litter, ranging from 2 months to over 2 years [Holmes, unpublished observations]. The five breeding pairs included here ranged from 3.5 to 6.5 months to produce their first litter. It would be interesting to test whether variability in the onset of endocrine changes corresponds with the variability in the time it takes a new pair of naked mole-rats to produce a litter.

Unexpectedly, we observed a small increase in *Cyp19a1* mRNA expression 1 week post-removal in females and a marked increase in females after the production of a litter. These increases were not seen in males and could be related to the dominant status the breeding female undertakes in the colony. Aromatase has been linked to aggression and dominance in other species. For example, Unger et al. [42] report that hypothalamic aromatase regulates aggression in both male and female mice, and Huffman, O’Connell, and Hofmann [43] report that an aromatase inhibitor decreases aggression in cichlid fish [42, 43]. Currently, little is known about how naked mole-rat female breeders maintain their dominance over others in the colony; therefore, it would be of interest to evaluate the role of aromatase given the increase in *Cyp19a1* during the transition to breeder status. Another possibility, which is not mutually exclusive, is that increased *Cyp19a1* mRNA could be related to female sexual behavior. Observations from *Cyp19a1* knockout mice suggest that aromatase may be necessary for female-typical sexual behaviors (for review, see [44]). Similarly in prairie voles, aromatase inhibitors reduce female sexual behaviors [45]. Regardless, dominance and sexual behaviors are both important for a naked mole-rat female to establish and maintain her position at the top of the social hierarchy. Evaluating the role that aromatase plays in social status and reproduction in naked mole-rats will shed light on key features of eusociality in mammals, including how breeding females suppress reproductive maturation in their colony mates. Given that aromatase converts testosterone to estradiol and we report low levels of circulating testosterone in all female groups, the increased *Cyp19a1* mRNA in breeding females could be associated with aromatase acting on other androgenic substrates. Alternatively, our single sampling paradigm might have missed cyclic variation in circulating androgens or reflect a dissociation between androgens in the blood and brain.

Finally, the group differences we see in body weight are consistent with previous reports that breeders, particularly queens, are often the largest animals in the colony (e.g., [9]). Furthermore, queen removal results in increased body weight of both male and female subordinates [14], suggesting the release from reproductive suppression triggers growth in non-reproductive animals. Indeed, new queens demonstrate remarkable vertebral lengthening associated with parity [46], which might also be associated with the release from reproductive suppression and representative of a pubertal growth spurt [47].

## Conclusions

The present study indicates that naked mole-rats exhibit sex-specific neuroendocrine changes when transitioning from subordination to reproductive maturation and social dominance. Naked mole-rats transitioning to breeding status resemble other mammalian species during puberty and begin to exhibit endocrine changes as early as 1 week following removal from the reproductive suppression of their colony. The sex-specific activation of gonadal steroid hormones and their receptors in the brain are clearly directly linked to reproduction. However, some of the neuroendocrine changes we report might also reflect sex-specific demands in colony living (e.g., dominance in breeding females and paternal behavior in breeding males). Given that reproductive hierarchies and large social colonies are two defining features of eusociality, these data reveal much about how this extreme form of social organization occurs in mammals. In conclusion, these data (a) confirm that naked mole-rats show sex differences in circulating gonadal hormones only when released from reproductive suppression, (b) demonstrate that latent sex differences in gonadal steroid hormone receptors exist in the brain of these remarkably monomorphic animals, and (c) provide future directions for evaluating the neuroendocrine basis of eusociality in naked mole-rats.

## Abbreviations

*Ar*: androgen receptor gene
AR: androgen receptor protein
*Cyp19a1*: aromatase gene
E2: 17β-estradiol
ELISA: enzyme-linked immunosorbent assay
*Esr1*: estrogen receptor alpha gene
LH: luteinizing hormone
P: progesterone
*Pgr*: progesterone receptor gene
T: testosterone
